# GSTM3 reverses the resistance of hepatoma cells to radiation by regulating the expression of cell cycle/apoptosis-related molecules

**DOI:** 10.3892/ol.2014.2358

**Published:** 2014-07-17

**Authors:** YING SUN, YU WANG, YUFENG YIN, XIANGHUA CHEN, ZHIJUN SUN

**Affiliations:** 1 Department of Internal Medicine, Shandong Medical College, Jinan, Shandong 250002, P.R. China; 2 Department of Gastroenterology, People’s Hospital of Rizhao, Rizhao, Shandong 276825, P.R. China; 3 The Health Education and Training Center of Shandong Province, Jinan, Shandong 250014, P.R. China

**Keywords:** GSTM3, cell cylce arrest, hepatocellular carcinoma, apoptosis, radioresistance

## Abstract

Radiotherapy (RT) is a major modality of hepatoma treatment. However, liver tumors often acquire radioresistance, which contributes to RT failure. The exact mechanisms of the radioresistance in hepatoma cells are largely unknown. Glutathione S-transferase M3 (GSTM3) is a phase II transferase, however, recent studies have suggested that GSTM3 is a potential tumor suppressor. The purpose of the present study was to investigate the role of GSTM3 in reversing radioresistance, and to explore the molecular mechanism of this in the human radiation-resistant PRF/PLC/5R hepatocellular carcinoma (HCC) cell line. The radioresistant PLC/PRF/5R cells were used as cell model, and were derived from PLC/PRF/5 parental cells using fractionated irradiation. The radiosensitivity of the cells was tested by clonogenic assay and flow cytometry analyses. The expression of B-cell chronic lymphocytic leukemia/lymphoma 2 (Bcl-2), Bax, p21, p27 and p53 was analyzed by quantitative polymerase chain reaction and immunoblotting with or without radiation. The results showed that the expression levels of GSTM3 were significantly lower in the PLC/PRF/5R cells than in the PLC/PRF/5 parental cells. GSTM3 overexpression sensitized the PLC/PRF/5R cells to radiation mainly though induction of apoptosis. According to the evidence from Annexin-V/PI staining, it markedly increased the percentage of apoptotic PRF/PLC/5R cells. The clonogenic assay indicated that GSTM3 significantly decreased the RT survival fraction in PRF/PLC/5R cells. Furthermore, GSTM3 increased the expression of cell cycle- and apoptosis-related genes (Bcl-2, Bax, p21, p27 and p53) in PRF/PLC/5R cells with irradiation. These findings suggest that GSTM3 plays an pivotal role in reversing the radioresistance of HCC and may be a potential target for sensitizing HCC cells to RT. The underlying mechanisms may be linked to the cell cycle arrest and apoptosis facilitation.

## Introduction

Hepatocellular carcinoma (HCC) is the fifth most common type of cancer and the third most frequent cause of cancer-related mortality worldwide. Over half a million new cases are diagnosed worldwide each year (1). Treatment of HCC consists of surgery with/without radiotherapy (RT) and chemotherapy. Ionizing radiation (IR) is a powerful modality of cancer treatment. For its advantage in preserving normal tissues, fractionated RT is often applied in preoperative treatment. However, hepatoma cells are prone to present radioresistant properties following irradiation, which are correlated with increased recurrence and therapeutic failure in numerous patients (2). PRF/PLC/5 HCC cells are derived from a primary hepatocellular carcinoma of an individual with positivity for serum hepatitis B virus surface antigen (HBsAg) (3). The PLC/PRF/5R cells of the present study were derived from PLC/PRF/5 cells, which show a higher radioresistance following irradiation treatment. As chronic hepatitis B virus (HBV) is the most common cause of HCC, this cell model is suitable for investigation of the exact molecular mechanisms underlying the adaptive resistance of HCC cells to IR.

Previous studies have suggested that glutathione S-transferases (GSTs), which have well-established roles in detoxification and clearance of ROS, have novel functions in pivotal signaling pathways for cell proliferation or apoptosis. For instance, GSTM1 functions as a negative regulator of MEKK1, which act as a upstream kinase in MAPK/P38 and JNK/ERK signal pathways (4). GSTP, another isoform of GSTs, has functions in ERK activation and JNK repression, which may account for the dual roles of GSTP in enhancement of growth inhibition and inhibition of apoptosis (5–7). GSTM3 has overlapping substrates with GSTM1, and GSTM genes are tandem-aligned on chromosome 1q13.3 (8). Thus, they may have similar functions as the signal transducer in key signal pathways. If GSTM3 is involved in regulating proliferation/apoptosis-associated pathways, it may potentially affect the radioresistance property in cells. As GSTM3 is always expressed at low levels in cancer tissues compared with that in normal tissues, it may possess tumor suppressive properties in tumor initiation and development (8–10). GSTM3 was also downregulated in radioresistant HCC cells according to the present findings. Therefore, the present study aimed to investigate the role of GSTM3 as a tumor suppressor and a novel target of radioresistance.

In order to identify whether GSTM3 is a potential therapeutic target to conquer the resistance in HCC, the current study explored the effects of GSTM3 overexpression on the radioresistance of PLC/PRF/5R cells to fractionated RT and the potential mechanism. The current study revealed a novel function of GSTM3 in regulating the adaptive radioresistance in HCC cells.

## Materials and methods

### Cell culture and transfection

Human HCC PLC/PRF/5R cell lines were purchased from American Type Culture Collection (Manassas, VA, USA). The HBsAg-producing cell line, PLC/PRF/5, was chosen for analyses. The radiation-resistant cell line, PLC/PRF/5R, was derived from fractional irradiated PLC/PRF/5 cells (six weeks of FR with total doses of 21 Gy). Cells were cultured in Dulbecco’s modified Eagle’s medium (Hyclone Laboratories, Logan, UT, USA) with 10% fetal calf serum (FCS; Gibco, Invitrogen, Grand Island, NY, USA), 100 units/ml penicillin and 100 μg/ml streptomycin at 37°C, 5% CO_2_ and 95% humidity. GSTM3 cDNA was inserted into a pcDNA3.1 vector (Invitrogen Life Technologies, Carlsbad, CA, USA) resulting in pcDNA3.1-GSTM3. The plasmids were transfected into PLC/PRF/5R cells using Lipofectamine 2000 (Invitrogen Life Technologies) to generate PLC/PRF/5R-con (pcDNA3.1/His-TOPO) cells and PLC/PRF/5R-GSTM3 (pcDNA3.1/His-TOPO-GSTM3) cells. The wild-type vector of pcDNA3.1/His-TOPO was used as a control. Stable cell lines were established by G418 screening. For the irradiation experiments, cells were treated with 2, 4, 6, 8 or 0 Gy (control) of X-ray using a linear accelerator (VARIAN 21EX 6 MV; Varian Medical Systems, Inc., Palo Alto, CA USA).

### Reverse transcription-quantitative polymerase chain reaction (RT-qPCR)

Cells were washed with phosphate-buffered saline (PBS) and homogenized in TRIzol reagent (Invitrogen Life Technologies). Total RNA was extracted and the concentration was determined on a Nanodrop ND-1000 spectrophotometer (Nanodrop, Wilmington, DE, USA). RNA (2 μg) was reverse-transcribed using a PrimeScript™ RT reagent kit (Takara Shuzo Co. Ltd., Dalian, China) according to the manufacturer’s instructions. GAPDH was used as a housekeeping gene, and the relative expression of the apoptosis and proliferation-related genes was detected using qPCR on a LightCycler 480 real-time instrument (Roche Diagnostics, Mannheim, Germany). The reactions were performed in a final volume of 20 μl containing 10 μl 2X SYBR Green Master mix (Takara Shuzo Co. Ltd.), 1 μl cDNA template and 0.25 μM each primer. The qPCR cycling parameters were as follows: 95°C for 30 sec; and 40 cycles of 95°C for 10 sec, 56°C for 25 sec and 72°C for 25 sec. At the end of the PCR, melting curve analyses of amplification products were carried out to confirm that only one product was amplified. The relative mRNA level of each gene was calculated as 2^−ΔΔCt^ (11). Differences between the PLC/PRF/5, PLC/PRF/5R-con and PLC/PRF/5R-GSTM3 groups were assessed by one-way analysis of variance. The primer sequences for GAPDH and proliferation/apoptosis-related molecules are listed in Table I.

### Western blot analysis

Cells were lysed in RIPA buffer plus protease inhibitors one day after irradiation, and protein was quantified by the bradford assay (Bio-Rad Laboratories, Inc., Hercules, CA, USA). In total, 20 μg protein of each group was separated by SDS-PAGE and transferred to a PVDF membrane. The protein bands were immunoblotted with the primary antibodies [rabbit polyclonal anti-human B-cell chronic lymphocytic leukemia/lymphoma 2 (Bcl-2), rabbit polyclonal anti-human Bax, mouse monoclonal anti-human p21, mouse monoclonal anti-human p27, mouse monoclonal anti-human p53 and rabbit polyclonal anti-human β-actin; 1:500; Cell Signaling Technology, Inc., Beverly, MA, USA] and secondary antibody (polyclonal goat anti-rabbit-HRP IgG; 1:1000; Pierce Biotechnology, Rockford, IL, USA). Following this, the bands were visualized by enhanced chemiluminescence substrate solution (GE Healthcare, Little Chalfont, UK) on a Syngene^TM^ gel imaging analysis system (Syngene, Cambridge, UK). β-actin was considered as a loading control.

### Cell proliferation assay

Cell viability was examined by Cell Counting Kit-8 (CCK-8; Dojindo, Kumamoto, Japan) according to the manufacturer’s instructions. Briefly, cells were plated at a density of 0.5×10^4^ cells/well in 96-well plates. Following incubation, cells were treated with 10 μl CCK-8 solution for 3 h. Absorbance was measured at 450 nm using a multiwell plate reader (Synergy HT, Bio-Tek, Winooski, VT, USA).

### Clonogenic assay

Twenty-four hours after irradiation, cells were plated on 9-cm dishes with 1,000 cells/well and maintained in culture for 10 days to alow colony formation. When visible colonies (at least 50 cells) emerged, they were fixed with 95% methanol, stained with 0.5% crystal violet and counted. Plating efficiency (PE) and surviving fraction (SF) were calculated as follows: PE = (colony number/seeded cell number) ×100%, SF = (PE_irradiated group_/PE_non-irradiated group_) ×100%. The cell survival curve was fitted using Origin 8.0 software (OriginLab Corporation, Northampton, MA, USA).

### Hoechst 33258 staining

Cells were washed with PBS and fixed using 4% paraformaldehyde for 30 min. Cells were then washed again and incubated in Hoechst 33258 solution for 10 min in the dark, at 37°C. Finally, the solution was removed, and cells were washed and observed with an inverted fluorescence microscope (Leica DMI300B, Leica, Wetzlar, Germany).

### Flow cytometric analysis of the cell cycle

A total of 1×10^6^ cells were trypsinized, washed and resuspended in 1 ml PBS with 5% FCS. Subsequently, 3 ml ice-cold ethanol was added to fix the samples. Prior to FCS analysis, samples were centrifuged at 1,500 × g, the supernatant was discarded and the cell pellet was resuspended in 1 ml PBS with propidium iodide (Sigma-Aldrich, St. Louis, MO, USA) and RNAse (Huamei, Shanghai, China) and incubated overnight at 4°C.

### Flow cytometric analysis of apoptosis

Cells were washed twice with ice-cold PBS, and then resuspended in binding buffer. Following this, cells were stained in Annexin V-fluorescein isothiocyanate (BD Biosciences, San Jose, CA, USA) and propidium iodide (Sigma-Aldrich), according to the manufacturer’s instructions. After a 30-min incubation in the dark at 37°C, cells were immediately examined on a FACSCalibur (BD Biosciences) flow cytometer and the data was analyzed with CellQuest software (BD Biosciences). All of the samples were tested in triplicate. The cell apoptosis rate was calculated following the formula: (N_apoptotic cells_/N_total cells_) ×100%.

### Statistical analyses

All experiments were independently repeated two or three times. The data are presented as the mean ± standard deviation. Statistical analyses were performed using SPSS 16.0 software (SPSS, Inc., Chicago, IL, USA). Values of P<0.05 were considered to indicate a statistically significant difference.

## Results

### GSTM3 is expressed at low levels in adaptive radioresistant HCC cells

**Figure 1:**
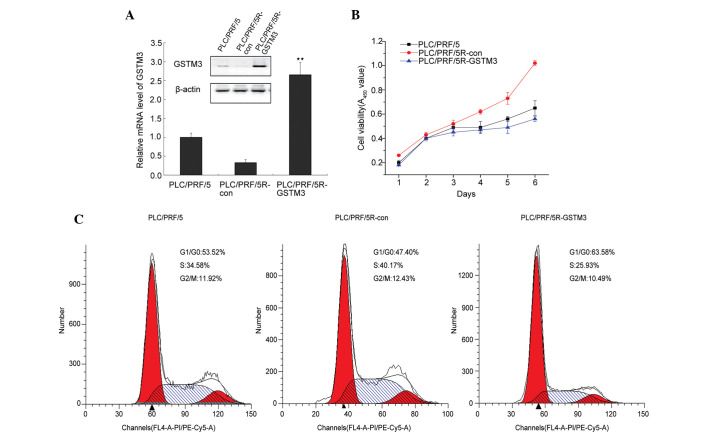
Overexpression of GSTM3 inhibits cell proliferation and induces cell cycle arrest in PLC/PRF/5R cells. (A) GSTM3 mRNA expression levels were analyzed in the PLC/PRF/5R-GSTM3 cells using reverse transcription-quantitative polymerase chain reaction and compared with those in the PLC/PRF/5 and PLC/PRF/5R-con cells. Error bars represent the SD. Western blot analysis confirmed GSTM3 over expression in PLC/PRF/5R-GSTM3 cells (data not shown). (B) Cell viability (represented by A_450_ value) was detected using the Cell Counting Kit-8 assay. Error bars represent the SD. (C) Cell cycle distribution was analyzed by propidium iodide staining. GSTM3, glutathione S-transferase M3.

The expression levels of GSTM3 were detected in the radiation-resistant cell line, PRF/PLC/5R, and the parental cell line, PRF/PLC/5. GSTM3 expression levels in the PRF/PLC/5 cells were three-fold greater than those in the PRF/PLC/5R cells. Additionally, GSTM3 protein expression levels, examined by western blotting, confirmed the RT-qPCR results (Fig. 1A). GSTM3 expression was also measured in the HepG3BR and HepG3B radioresistant and radiosensitive HCC cell lines: GSTM3 expression levels were significantly lower in the radioresistant types (data not shown). These results suggest that low levels of GSTM3 expression may be associated with the adaptive radioresistance of HCC.

### GSTM3 overexpression inhibits the growth of PRF/PLC/5R cells

**Figure 2:**
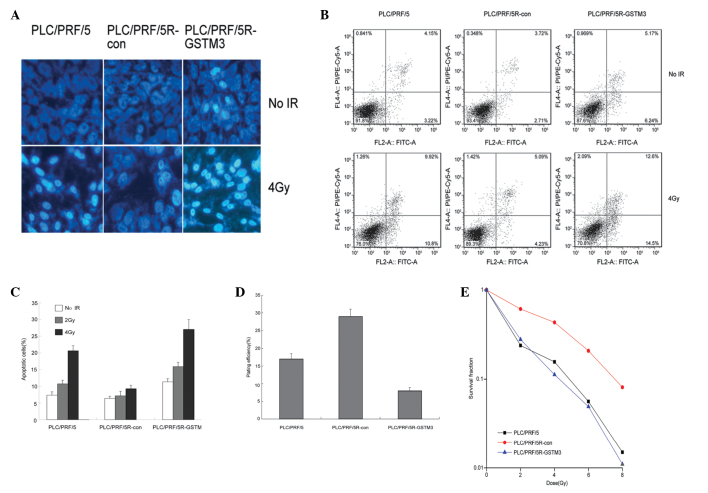
Overexpression of GSTM3 reverses the radioresistance of PRF/PLC/5R cells. (A) Hoechst 33258 staining represents the morphological changes of different cells following irradiation (magnification, ×200). Apoptotic cells were distinguished by characteristic bright blue fluorescence of nuclei due to condensed or fragmented chromatin. Representative images were acquired with a Leica inverted fluorescence microscope. (B) The hepatocellular carcinoma cells were exposed to 0 or 4 Gy ionizing radiation and examined by Annexin V/propidium iodide staining two days following irradiation. (C) Numbers of apoptotic cells were counted two days following exposure to 2 and 4 Gy IR. Error bars represent the SD. (D and E) Cells were incubated in six-well plates for 24 h and then radiated. The colonies were counted after 10 days. The plating efficiency (D) and surviving fraction (E) were calculated as indicated. GSTM3, glutathione S-transferase M3.

To investigate the role of GSTM3 in the radiosensitivity of PRF/PLC/5R cells, a GSTM3-overepressing vector was transfected into PRF/PLC/5R cells to construct a cell line that stably expressed GSTM3 (PRF/PLC/5R-GSTM3). This showed a nine-fold increase in GSTM3 transcript levels in contrast to PRF/PLC/5R-con cells, which was confirmed by immunoblotting (Fig. 1A). As GSTM1 plays a role in inhibiting the activity of proliferation regulator MEKK (4), we proposed that it may act as a transducer in tumor growth signaling pathways. Studies have reported low GSTM3 expression in several types of tumors (8–10), and this has been found to be associated with cisplatin resistance in breast cancer (10). Therefore, we hypothesized that GSTM3 may be similar to GSTM1, and act as a signal transductor in proliferation-related signaling pathways. We first examined the effect of GSTM3 on cell proliferation. The CCK-8 assay showed that GSTM3 dramatically inhibited the cell proliferation of PRF/PLC/5R cells. The cell viability of PLC/PRF-5R-GSTM3 was less than that of the PLC/PRF/5R-con at day four (P<0.05; Fig. 1B). Cell cycle analysis indicated that GSTM3 induced G0/G1-phase arrest and subsequently a decreased number of cells in the S-phase compared to the control PRF/PLC/5R-con and PRF/PLC/5 cells (Fig. 1C). Meanwhile, the apoptotic cell number increased to 11.4% in the PRF/PLC/5R-GSTM3 cells (Fig. 2A and C).

### GSTM3 overexpression sensitizes PLC/PRF/5R cells to irradiation

To assess whether GSTM3 overexpression could reverse the radioresistance of the PLC/PRF/5R cell line, we assessed the role of GSTM3 on cell apoptosis in radiated PLC/PRF/5R cells. As cell death by apoptosis may occur following IR, we first used Hoechst33258 staining to measure the morphological changes of apoptotic HCC cells induced by IR (Fig. 2B). Cells with characteristic bright blue fluorescent nuclei were defined as apoptotic cells, as the condensed chromatin of apoptosis cells was strongly stained. As shown in Fig. 2B, apoptotic cells in the PLC/PRF/5 and PLC/PRF/5R-GSTM3 groups were dramatically increased compared with those of the radioresistant PLC/PRF/5R-con group at 4 Gy IR. The Annexin V/PI staining assay was conducted to determine the apoptotic cell number induced by irradiation. PLC/PRF/5 cells were sensitive to irradiation; the apoptotic rate increased from 7.37 to 20.7% post-IR. The PLC/PRF/5R-con cells were resistant to irradiation; the apoptotic rate increased from 6.43 to 9.32% after 4 Gy IR. Although it increased, this difference was not significant, and therefore, no significant difference was identified in the levels of apoptosis post-IR. GSTM3 overexpression significantly increased the apoptotic rate from 11.41 to 27.1% in the PLC/PRF/5R-GSTM3 group (Fig. 2A). Similar data were observed in the 2-Gy irradiated HCC cells (Fig. 2C). The effect of GSTM3 on the radiation response of PLC/PRF/5R cells was also evaluated using a clonogenic assay. GSTM3 overexpression decreased the PE of PLC/PRF/5R cells to 8% in contrast to the control PLC/PRF/5R-con cells to 29% (Fig. 2D), and increased the radiosensitivity of PLC/PRF/5R cells, as shown by the notable reduction in SF of clonogenic cells at 2, 4, 6 and 8 Gy (SF of PLC/PRF-5R-GSTM3 vs. PLC/PRF-5R-con; P<0.05). The sensitization enhancement ratio was 1.5 (Fig. 2E).

### GSTM3 overexpression affects the expression of cell cycle/apoptosis-related proteins

**Figure 3:**
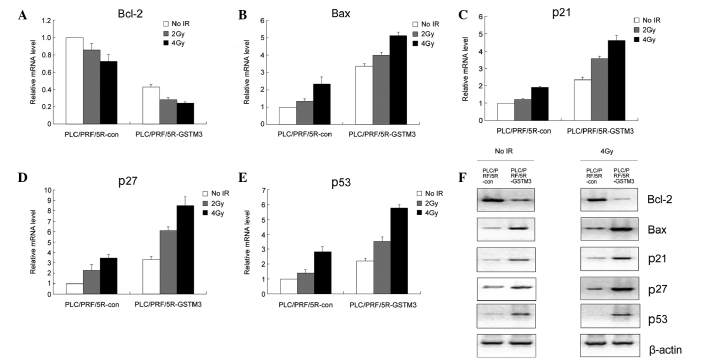
GSTM3 transfection affects the expression of cell cycle/apoptosis-related proteins in PRF/PLC/5R cells exposed to 2 or 4 Gy IR. (A–E) Total RNA was harvested one day after irradiation. The mRNA levels of Bcl-2 (A), Bax (B), p21 (C), p27 (D) and p53 (E) were measured by reverse transcription-quantitative polymerase chain reaction and normalized by GAPDH. Error bars represent the SD. (F) Western blot analysis of Bcl-2, Bax, p21, p27 and p53 in PRF/PLC/5R-Con and PRF/PLC/5R-GSTM3 cells treated with 0 or 4 Gy irradiation. Representative blots are shown, and β-actin was used as the loading control. GSTM3, glutathione S-transferase M3; Bcl-2, B-cell chronic lymphocytic leukemia/lymphoma 2.

As abovementioned, GSTM3 overexpression may be correlated with the arrest of cell cycle progression and potentiation of apoptosis in PLC/PRF/5R cells. Therefore, the expression of a number of molecules involved in these two processes was analyzed. First, RT-qPCR was used to compare the mRNA expression levels of Bcl-2, Bax, p21, p27 and p53. GSTM3 overexpression markedly decreased the Bcl-2 expression and increased the Bax, p21, p27 and p53 expression in contrast to that in the PLC/PRF/5R-con cells. Following irradiation, the expression of Bax, p21, p27 and p53 were elevated, but GSTM3 overexpression induced markedly higher expression levels of these molecules compared with those in the control cells (Fig. 3A–E). Data regarding the protein expression levels of Bcl-2, Bax, p21, p27 and p53 at 0 and 4 Gy also confirmed the results of the RT-qPCR (Fig. 3F).

## Discussion

While RT provides useful palliation on HCC, advanced HCC remains incurable, as the previously sensitive tumor rapidly becomes resistant to IR. The acquired resistance may arise from tolerance of stimuli, decreased transducer activity, inactivation of normal cell death, decreased pro-apoptotic factors, altered cell cycling factors and proliferation signals (12–14). Therefore, there is an urgent requirement for the identification of novel biomarkers which may predict the prognosis and help to select the patients that will benefit from RT. Furthermore, elucidating the underlying molecular mechanisms of the radioresistance of HCC cells is important for the development of novel target agents for radiation sensitization, and for exploring optimal radiotherapeutic regimens.

GSTs are members in gene superfamilies of the phase II detoxification enzymes. GSTs can be classified into four categories on the basis of sequence homology and chromosomal localization, namely α, μ, π and θ (15). GSTM3 is a GST-Mu class member, which is known to be involved in regulating the susceptibility to cancer (8,16–19). However, little is known regarding the role of GSTM3 in antitumorigenesis or IR sensitization.

To the best of our knowledge, this is the first study to anlayze the novel effect of GSTM3 on RT sensitization. The results showed that GSTM3 overexpression significantly increased the cell apoptosis rate and apoptosis-related gene expression in PLC/PRF/5R cells, at the base level and after IR. Bcl-2 and Bax both are members of Bcl-2 family. The former is an anti-apoptosis protein and the latter is a pro-apoptosis protein (20). In the present study, overexpression of GSTM3 downregulated the Bcl-2 expression and upregulated the Bax expression. This was consistent with the effects of GSTM3 in promoting the apoptosis of radioresistant PLC/PRF/5R cells. In addition, p21, p27 and p53 expression was increased in GSTM3-overexpressing cells. p21 and p27 are famous cyclin-dependent kinase inhibitors. They are members in the Cip/Kip family, which can arrest the cell cycle and serve as tumor suppressors (21). Cell cycle arrest contributes to proliferation inhibition and apoptosis induction. In the present flow cytometric assay, GSTM3 overexpression was shown to cause G0/G1 arrest in PLC/PRF/5R cells. Lamore and Wondrak (22) and Cabello *et al* (23) reported that GSTM3 upregulation is coordinated with an increase in p21 and p53 expression. p53 is a pivotal molecule in HBV-caused HCC for its transcriptional suppression on HBx (24). GSTM3 may act as a tumor suppressor and overcome the effect of HBx in inhibiting p53 transcription. According to the present clonogenic assay results, GSTM3 overexpression increased the radiosensitivity of PLC/PRF/5R cells and decreased the SF of clonogenic cells at 2, 4, 6 and 8 Gy. In addition, the expression of Bax, p21, p27 and p53 were markedly higher following irradiation in the GSTM3-overexpressing cells. Overall, GSTM3 regulation of apoptosis- or proliferation-related pathways may contribute to the effects of GSTM3 in reversing the radioresistance of HCC cells.

In conclusion, the results of the present study suggest that GSTM3 is not only a enzyme in phase II biotransformation, but also has novel functions in irradiation sensitization of HCC cells. GSTM3 regulates the expression of cell cycle/apoptosis-related proteins and, thus, inhibits the proliferation and reverses the radioresistance of PLC/PRF/5R cells. Therefore, previously discovered enzymes may exert effects other than their superficial functions. This study provides promising prospects for novel radiotherapeutic regimens acheiving more benefits from fractionated RT. However, the exact molecular mechanisms of GSTM3 in sensitizing cells to IR and the optimal target sensitizer in HCC require further investigation.

## Figures and Tables

**Table I:** Primers used in reverse transcription-polymerase chain reaction.

Gene name	Forward primers (5′-3′)	Reverse primers (5′-3′)	Fragments (bp)
GAPDH	TGCCGTCTAGAAAAACCTGC	ACCCTGTTGCTGTAGCCAAA	485
Bcl-2	GTGGAGGAGCTCTTCAGGGA	AGGCACCCAGGGTGATGCAA	368
Bax	AAGAAGCTGAGCGAGTGT	GGAGGAAGTCCAATGTC	462
p21	CACCCTAGTTCTACCTCAGGCA	ACTCCCCCATCATATACCCCT	412
p27	ACGGGAGCCCTAGCCTGGAGC	TGCCCTTCTCCACCTCTTGCC	500
p53	TGGCCATCTACAAGCAGTCACA	GCAAATTTCCTTCCACTCGGAT	375

Bcl-2, B-cell chronic lymphocytic leukemia/lymphoma 2.
